# Structural characterization, antioxidant activity and anti-inflammatory of the phosphorylated polysaccharide from *Pholiota nameko*

**DOI:** 10.3389/fnut.2022.976552

**Published:** 2022-08-31

**Authors:** Xu Zhang, Tingting Liu, Xi Wang, Lanying Zhou, Ji Qi, Siyu An

**Affiliations:** ^1^College of Food Science and Engineering, Jilin Agricultural University, Changchun, China; ^2^Jilin Province Product Quality Supervision and Inspection Institute, Changchun, China

**Keywords:** *Pholiota nameko* polysaccharide, high-temperature pressurized extraction, structural characterization, anti-inflammatory, antioxidant

## Abstract

In this study, a novel polysaccharide (SPN) was extracted by high-temperature pressure method and purified by a DEAE-52 column and a Sephadx G-100 gel column. PPN was obtained after phosphorylation of SPN. The differences of structural features, antioxidant activity, and anti-inflammatory effect of the two polysaccharides were investigated by chemical methods and RAW 264.7 cell model. SPN (Mw = 15.8 kDa) and PPN (Mw = 27.7 kDa) are an acidic polysaccharide with β-pyranose configuration, mainly containing rhamnose, mannose, glucose, arabinose, and galacose. FI-IR, NMR, and SEM spectra showed phosphorylation of SPN changed its structure. In methylation analysis, the major chains of SPN and PPN were 1,4-linked Glc*p*, 1,6-linked Gal*p*, 1,2-linked Rha*p*, and 1.6-linked Man*p* with terminals of t-linked Glc*p*, t-linked Ara*f*. The side chain of SPN was 1,4,6-linked Gal*p*, 1,2,5-linked Ara*f*, while the side chain of PPN was 1,4,6-linked Gal*p*, 1,2,4-linked Glc*p*. In antioxidant activity experiments, the free radical scavenging rate of PPN was stronger than that of SPN. Also, PPN always has better anti-inflammatory on RAW 264.7 cells induced by LPS than that of SPN in same concentration, and it plays an anti-inflammatory role by inhibiting PI3K/AKT/mTOR pathway. The results indicated polysaccharide could significantly improve its antioxidant and anti-inflammatory function after phosphorylation. This study provides a potentially antioxidant and anti-inflammatory health food and drug.

## Graphical Abstract

Structure and bioactivity of phosphorylated *pholiota nameko* polysaccharide. 
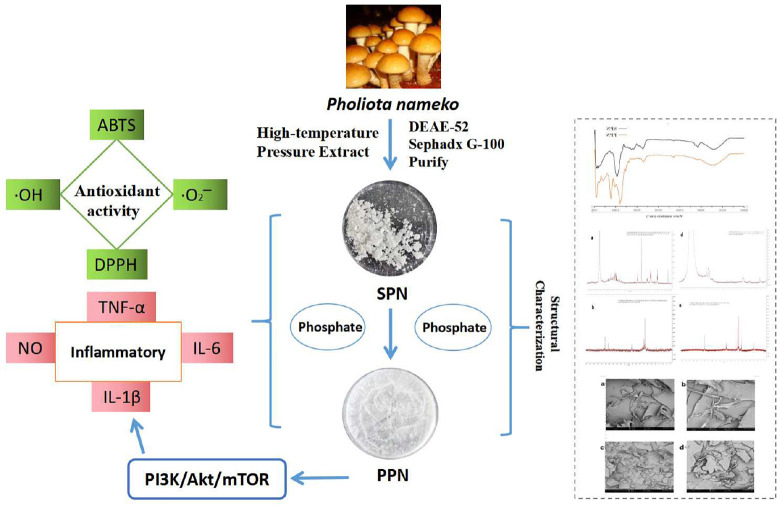

## Introduction

Inflammation is a normal immune defense mechanism of the body, but it is a double-edged sword. The short-term clearance of inflammatory by the defense system can promote the recovery of the body. However, the long-term inflammatory defense could lead to tissue organs damage, causing anaphylactic reactions, or even disease (1). When immune response is stimulated by pathogens, the macrophages secret proinflammatory cytokine (2). Excessive production of inflammatory cytokines could lead to fever, asthma, arthritis, and even cancer (3). Free radicals widely exist in the human body and are important factors in regulating cellular signaling pathways and maintaining body balance (4). Oxidative stress is caused by excessive free radicals, leading to cardiovascular disease (5), inflammatory (6), immunomodulatory (7), hypertension, and even tumor (8).

Edible fungi are mushrooms with edible fruit body and have a long research history in China (9). *Pholiota nameko*, one kind of edible fungi, belongs to genius *Lepidoptera* (Cortinariaceae). It originated in Japan and has distributed in many provinces of China (10). This kind of mushroom can be easily cultivated and is regarded as a healthy food with higher carbohydrate and lower fat. Polysaccharides extracted from edible fungi are normally biological compounds which have functions of anticancer (11), anti-inflammatory (12), antioxidant (13), hypoglycemic (14), intestinal regulation (15), and immunization (16, 17). The chemical composition, structural characteristics, and biological activity of polysaccharides are affected by different extraction methods (18). High-temperature pressure method is a green extraction method with few solvents and low energy consumption. It uses high temperature and high pressure to increase the solubility of target compounds and solvent diffusion rate. Studies have shown that the extraction rate of polysaccharide by high-temperature pressure method was higher than that by microwave, ultrasonic, and hot water extraction methods (19, 20). Lee et al. (21) have shown that polysaccharide with higher molecular weight have better anti-cancer effects. Therefore, the high temperature and pressure method is an effective green extraction method to improve the extraction rate and biological activity of polysaccharide. Moreover, in order to enhance biological function of natural polysaccharides, some methods are used to modify their structure (22). There exist several ways for structure modification, such as chemical methods, physical methods, and biological methods. Among these methods, chemical methods are the most widely adoption (23). Chemical modification can improve or change biological functions of polysaccharides mainly through changing molecular weight and type, position and number of substituents in structure of polysaccharides (24). The principal methods of chemical modification are complexation with metal ions, sulfation, sulfonylation, acetylation, alkylation, selenization, carboxymethylation, phosphorylation, and benzoylation. Phosphorylation of polysaccharides is modified by introducing phosphate ester bonds into structure of polysaccharide (25). Since phosphate has three negative charges, it can affect certain activities of polysaccharides, like antioxidant activity, anticancer activity by enhancing their electronegativity (26, 27). However, few researches have investigated structures and functions of polysaccharides from fruit body of *P. nameko* and phosphorylated modification of these polysaccharides.

In this study, a novel polysaccharide was extracted from dried body of *P. nameko* and purified by a DEAE-52 column and a Sephadx G-100 gel column. Its structure features, antioxidant activity, and anti-inflammatory effects were determined by chemical methods and RAW267.4 cells. Also, structural and functional comparison between this novel polysaccharide and its phosphorylated polysaccharide were investigated. This study could provide theoretical bases in further researches about a potential anti-inflammatory and antioxidant activity from phosphorylated mushroom polysaccharide.

## Materials and methods

### Materials and reagents

*Pholiota nameko* was cultivated in Panshi City, Jilin Province, China, and purchased from the Zhongdong Market in Changchun City, Jilin Province, China. DEAE-52 cellulose was bought from Solarbio Science and Technology Co., Ltd. (Beijing, China) and Sephadex G-100 was purchased from Shanghai Yuanye Biotechnology Co., (Shanghai, China). LPS and standard monosaccharides (rhamnose, mannose, glucose, xylose, galactose, and arabinose) were acquired from Sigma Chemical Co. All other chemical reagents were analytical grade from Beijing Chemical Works.

### Polysaccharides preparation

#### Extraction of crude polysaccharide

According to High-temperature pressurized extraction (28), 100 g dried mushroom mixed with 900-mL distilled water for 12 h at room temperature (25^°^C). An autoclave (U1000, Xinyi CO., Shanghai, China) was used for extraction of crude polysaccharide (CPN) from the mixture at 110^°^C, 1.2 MPa for 30 min. After centrifugation, concentration, and deproteinization (Savge method), solution of CPN was obtained. Adding four times ethanol into the solution to stand for 10 h at 4^°^C then centrifugating at 4,500 rpm for 10 min and lyophilizating for 24 h, crude polysaccharide was obtained.

#### Isolation and purification of novel polysaccharide

**FIGURE 1:**
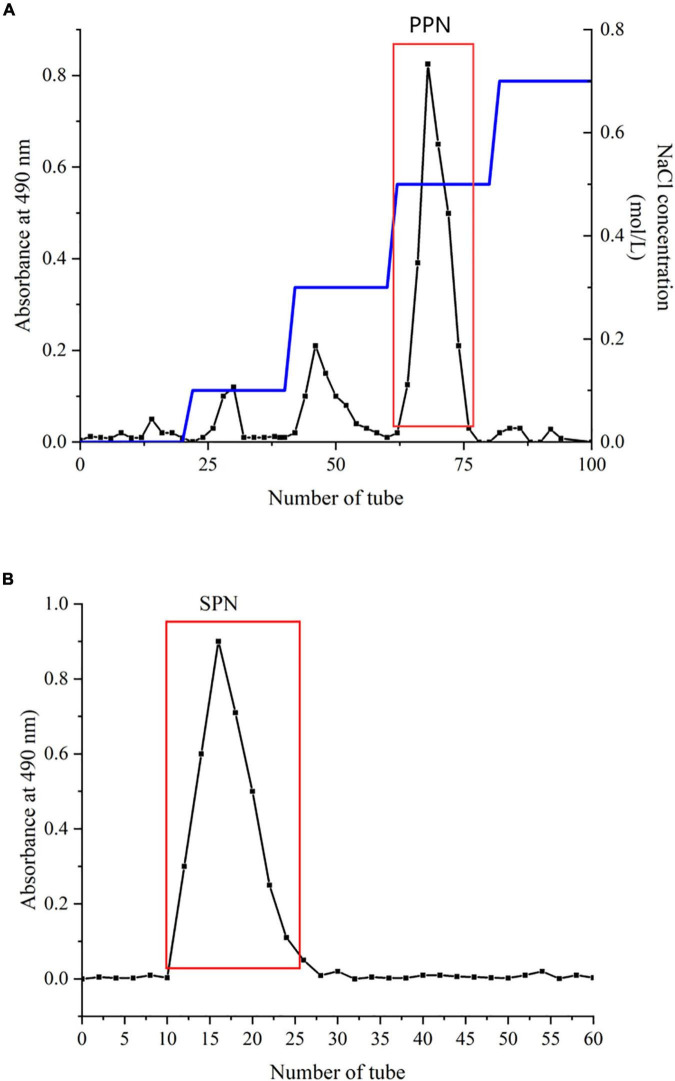
Chromatography of SPN on DEAE-52 **(A)** and Sephadex G-100 **(B)**.

The method was determined by Zhou et al. (29) with slight modification. A DEAE-52 column (2.6 × 40 cm) and a Sephadx G-100 gel column (1.6 × 40 cm) were used for separation and purification (30). Briefly, 1 g CPN was dissolved in deionized water (1:20, *w/v*), centrifuged at 4,500 rpm for 10 min. Then, the supernatant was loaded in a DEAE-52 column, flowed with different concentrations of NaCl solution at 1.0 mL/min. The effluents were collected and measured by phenol sulfuric acid method. The main fractions are showed in Figure 1A. For purification, each fraction was subsequently injected in a Sephadx G-100 gel column, eluted by deionized water at 0.5 mL/min. The fraction of the most symmetrical eluting peak was collected (Figure 1B). After concentration, dialyzation, and lyophillation, the purified polysaccharide (SPN) was obtained for further study.

#### Phosphorylation and degree of substitution of phosphorylated polysaccharide

Phosphorylation of SPN was based on method of Xiong et al. (31). Three gram sodium triphosphate and 1 g sodium trimetaphosphate were dissolved in 100-mL distilled water. Then, 500 mg SPN and 2.5 g sodium sulfate was added into this solution. After adjusting pH to 8.5 by NaOH (5 mol/L), the solution was placed at a water bath (80^°^C) and then dialyzed for 48 h in a dialysis bag (MwCO 8,000–14,000 Da). Adding four times ethanol into this solution for 12 h at 4^°^C then centrifugating at 4,500 rpm for 10 min and lyophilizating for 24 h, phosphorylated polysaccharide (PPN) was obtained. The degree of substitution is calculated by following formula (32):


(1) $$\text{DS}=\frac{5.23\,P}{100-3.32\,P}$$

P, percentage of phosphorus element; DS, degree of phosphate substitution.

### Chemical analysis

The total sugar content of SPN and PPN were measured by phenol-sulfuric acid method (33). The Bradford method was used to measure the protein content of SPN and PPN (34). The uronic acid content of SPN and PPN was determined by the sulfuric acidcarbazole method (35).

The functional groups of SPN and PPN powders in KBr pellets were analyzed by a RESTIGE-21 IR spectrometer (Shimadzu, Japan) at range of 4,000–400 cm^–1^.

The homogeneity and molecular weights of SPN and PPN were determined by Ultrahydrogel 2000 (12 μm, 7.8 mm × 300 mm) series Ultrahydrogel 500 (10 μm, 7.8 mm × 300 mm), using an alliance 2,695 gel permeation chromatography (Waters, Singapore).

Monosaccharide composition of SPN and PNP was investigated using a UltiMate 3000 high-performance liquid chromatography equipped with photo-diode-array-detector (Thermo, Germany) according to Yang et al. (36).

### Methylation analysis

A method reported by Ji et al. (37) was used for methylation analysis of SPN and PPN with modification. In brief, 5 mg of sample was dissolved in 1-mL distilled water, then the solution was added 200 μL of 0.2 mol/L MES [2-(4-Morpholino) ethanesulfonic] solution and 400 μL of 0.5 mg/L carbodiimide solution. After reaction for 3 h at 25–30^°^C, 1 mL of 4 mol/L imidazole-HCL solution and 600 μL of 70 μg/L NaBD_4_ was added into the solution for overnight reaction at 4^°^C. Afterward, 500 μL ethylic acid was used to stop the reaction. Then, the reduced sample was obtained after dialysis (MWCO 3,500 Da) and lyophilization.

After reduction, 5 mg sample dissolved in 0.5 ml of NaOH/DMSO and methylated with 1 mL CH_3_I. After two times of methylation, the OH absorption band around 3,400 cm^–1^ was disappeared determined the methylation was completed. The methylated sample was added with 1 mL trifluoroacetics acid (2 mol/L) at 120^°^C for 3 h. After removing the trifluoroacetic acid by absolute ethanol, methylated sample was reduced by sodium borohydride (30 μg/L) at room temperature for 12 h. Later, this sample was acetylated by pyridine and acetic anhydride and dissolved in dichloromethane for GC-MS analysis. The methylation result was determined by GC-MS (7890B-5977 Agilent, United States) equipped with a column of Agilent DB-35 ms (30 m × 0.25 mm × 0.25 μm) and an ion trap MS detector. Its temperature program was as follows: 140^°^C for 1 min; raised to 170^°^C at a rate of 10^°^C/min, maintained 2 min; raised to 180^°^C at a rate of 2^°^C/min, maintained 3 min; raised to 220^°^C at a rate of 4^°^C/min, maintained 1 min; raised to 280^°^C at a rate of 20^°^C/min, maintained 5 min. Helium (0.8 mL/min) was used as the carrier gas.

### Nuclear magnetic resonance spectroscopy

^1^H, ^31^P, and ^13^C NMR spectra of the SPN and PPN were recorded on an Avance-600 NMR spectrometer (Bruker, United States).

### Scanning electron microscope analysis

SPN and PPN were gold-plated and observed by a SEE-550 SEM (Shimadzu, Japan) with accelerating voltage of 5 kV and amplification of 1,000× and 5,000×.

### Assay of antioxidant activity

#### Assay of DPPH radical scavenging

DPPH (1,1-diphenyl-2-picrylhydrazyl) radical scavenging of SPN and PPN was based on method of Ji et al. (38). The reaction solution composed of 0.5 mL sample solution with different concentrations and 2.0 mL of 0.2 mol/L DPPH solution. The mixture reacted in a dark room at 30^°^C for 30 min and the absorbance was surveyed at 517 nm. Vitamin C (Vc) was used as a positive control. The equation for calculating radical removal activity was as following:


(2) $$\mathrm{DPPH}\mathrm{scavenging}\mathrm{rates}\left(\%\right)=\frac{\mathrm{Do}-\mathrm{Di}}{\text{Do}}\times 100\%$$

where Do was the blank solution absorbance (distilled water instead of the sample), Di was the sample solution absorbance.

#### Assay of ABTS radical scavenging

ABTS [2,2′-Azinobis-(3-ethylbenzthiazoline-6-sulphonate)] radical removal rate was determined as previously reported methods with minor modifications (39). The ABTS premix prepared from 10 mL of 2.4 mmol/L ABTS and 10 mL of 7 mmol/L potassium persulfate was kept in dark room for 12 h. The ABTS reaction solution was prepared by diluting the premixed solution with water to the absorbance of 0.700 ± 0.02 at 734 nm. 3 mL sample solution and 3 mL ABTS reaction solution were mixed and retained in a dark room for 10 min. The absorbance was analyzed at 517 nm. Vc was used as a positive control. The equation for calculating radical removal activity was as following:


(3) $$\mathrm{ABTS}\mathrm{scavenging}\mathrm{rates}\left(\%\right)=\frac{\mathrm{Ao}-\mathrm{Ai}}{\text{Ao}}\times 100\%$$

where Ao was the blank solution absorbance (distilled water instead of the sample), Ai was the sample solution absorbance.

#### Assay of hydroxyl radical scavenging

For hydroxyl radical scavenging assay, 1 mg of sample solution added with 1 mL of 9 mol/L FeSO_4_, 1 mL of 9 mmol/L salicylic acid, and 1 mL of 6 mmol/L H_2_O_2_, and reacted at 37^°^C for 30 min. The absorbance of the sample solution was determined at 510 nm. Vc was used as a positive control. The equation for calculating radical removal activity was as following:


(4) $$\mathrm{Hydroxyl}\mathrm{scavenging}\mathrm{rates}\left(\%\right)=\frac{\mathrm{Ao}-\mathrm{As}}{\text{Ao}}\times 100\%$$

where Ao was the blank solution absorbance (distilled water instead of the sample), As was the sample solution absorbance.

#### Assay of superoxide anion radical scavenging

For superoxide anion radical scavenging assay, the 0.5 mL sample solution added to 2.5 mL of 50 mmol/L Tris-HCl buffer (pH 8.2) and incubated at 30^°^C for 30 min. After 0.5 mL of 5 mmol/L pyrogallol was added to the incubated solution, the mixture was shaken quickly, and the absorbance of the reaction solution was determined at 325 nm. Vc was used as a positive control. The equation for calculating radical removal activity was as following:


(5) $$\mathrm{Superoxide}\mathrm{anion}\mathrm{scavenging}\mathrm{rates}\left(\%\right)=\frac{\mathrm{Uo}-\mathrm{Uc}}{\text{Uo}}\times 100\%$$

where Uo was the blank solution absorbance (Tris-HCl buffer instead of the sample), Uc was the sample solution absorbance.

### Assay for inflammatory effect on RAW 264.7 cells

#### Cell culture

The RAW 264.7 cells from mice were purchased by ATCC. The cells were cultured in a H-DMEM medium (Hyclone, United States) with 10% FBS (Biological Industries, Israel) and 1% penicillin-streptomycin at an 37°C incubator with humidified atmosphere of 5% CO_2_. RAW 264.7 cells of 3–15 passages were used in this study.

#### Cell viability analysis

A method reported by Zhuang et al. (40) was used for cell viability analysis with modification. Cell Counting Kit-8 (CCK-8, Bioss Co., Beijing, China) was used to evaluate the cell viability. Briefly, RAW 264.7 cells were cultured in 96-well plates with a density of 5 × 10^3^ cells/mL at a 37^°^C incubator with humidified 5% CO_2_ atmosphere for 4 h. Then, the cells were treated with different concentrations of SPN (50, 100, 200, 300 μg/mL) and PPN (50, 100, 200, 300 μg/mL) for 24 h. After that, 10 μL of CCK-8 solution was added and these cells were incubated at the same conditions for 2 h. The cell viability was determined by a multifunctional enzyme marker (Thermo Fisher, United States) at a wavelength of 450 nm and was expressed as a relative percentage to the blank control group.

#### NO concentration analysis

The 1.5 × 10^5^ cells/mL of RAW 264.7 cells were cultured in a 96-well plate at 37^°^C and 5% CO_2_ incubator for 24 h, then treated with 100 μL of 1 μg/mL LPS or 100 μL of different concentrations of SPN (100, 200, 300 μg/mL) + LPS and PPN (100, 200, 300 μg/mL) + LPS for 24 h. NO concentration was measured by Griess Reagent method. 100 μL supernate of every well was added with 50 μL sulfanilamide solution (100 g/L) and 50 μL amines-phosphoric acid solution (0.1 g amines dissolved in 10 mL 5% phosphoric acid solution). The absorbance was measured by a multifunctional microplate (Thermo Fisher, United States) reader at wavelength of 540 nm. 1 μg/mL LPS was used as positive control.

#### Assay of TNF-α, IL-1β, and IL-6 levels

RAW 264.7 cells (1.5 × 10^5^ cells/mL) were incubated in 96-well plate for 24 h. Afterwards, the cells were treated with 1 μg/mL LPS or different concentrations of SPN (100, 200, 300 μg/mL) + LPS and PPN (100, 200, 300 μg/mL) + LPS for 24 h. The TNF-α, IL-1β, and IL-6 levels were measured by related ELISA kits (MultiSciences Co., China). 1 μg/mL LPS was used for positive control.

#### Assay of mRNA expression levels

The mRNA expression levels were determined by quantitative RT-PCR according to the method of Sun et al. (41). Briefly, RAW 264.7 cells were treated were treated with 1 μg/mL LPS or different concentrations of PPN (100, 200, 300 μg/mL) + LPS for 24 h. And the treated cells were collected for total RNA extraction. cDNA was synthesized by transcription kit (TaKaRa Co., China). The expression levels of PI3K, Akt, and mTOR were analyzed by a quantitative real-time PCR instrument (Roche, Swiss). The mRNA expression levels were calculated by 2^–ΔΔ*CT*^ method with β-actin as internal reference gene. The primer design sequences of real-time PCR are shown in Table 1.

**TABLE 1:** The primer sequence of real-time PCR.

Primers	Forward	Reverse
β-actin	5′-CGTAGCTAGCTAGCTAGCTAGCTC-3′	5′-ACTAGCTAGCTAGTCGATCGTACG-3′
PI3K	5′-ATCGATCGATCGTAGCTAGCTCGA-3	5′-CGTACGTAGCTAGCTAGCTAGCTG-3′
Akt	5′-GTAGCTACTAGCTATCAGTCATCGT-3′	5′-CGTAGCTAGCTAGCTAGCTGATCGC-3′
mTOR	5′-AGTCGATCGTACGTAGCTGATGCT-3′	5′-CGTACTACGTACGATCGTGTACGA-3′

### Assay of protein expression levels

The RAW 264.7 cells were collected after treatment by polysaccharides. The cells were lysed by RIPA and the whole protein lysate was collected. And the protein concentration was determined using the BCA Protein Detection Kit (Solarbio Co., China). The loaded amount of the protein was 10 μg in each experiment. The protein was separated by SDS-PAGE and transferred to PVDF membrane. Then, PVDF membrane was blocked with TBST blocking solution for 1 h. The diluted primary antibody was incubated for 12h at 4°C. The diluted secondary antibody was incubated at room temperature for 1h. The protein expression levels of the p-PI3K, p-Akt, and p-mTOR were detected by gel Imager (Tianneng, China).

### Data analysis

All the test data of this study were expressed as means ± SD (no fewer than triplicate determinations) and analyzed with variance (ANOVA) followed multiple tests. SPSS V22.0 was used to all statistical analysis and *p* < 0.05 was considered to be significant.

## Results and discussion

### Extraction and purification

SPN was extracted from dried mushrooms by high-temperature pressurized method and purified by a DEAE-52 column (2.6 × 40 cm) at mobile phase of 0.5 NaCl solution and a Sephadx G-100 gel column (1.6 × 40 cm) with ultrapure water. The yield of CPN was 41.2% relative to dried mushroom and the yield of SPN was 35.3% relative to CPN.

After phosphorylation of SPN, phosphorylated polysaccharide (PPN) was obtained. The DS of PPN was 0.52 which is similar with other phosphorylated polysaccharides as previous research reported (42).

### Chemical analysis

From the phenol-sulfuric acid measurement, the sugar content of SPN and PPN were, respectively, 96.5 and 83.4%. The uronic acid of SPN was 21.3%, while the uronic acid of PPN was 24.7%. Also, their protein contents were separately 1.02 and 0.87%. After phosphorylation, the sugar content decreased, probably resulting from the loss during phosphorylation, which was similar to the results reported by previous researches (43).

**FIGURE 2:**
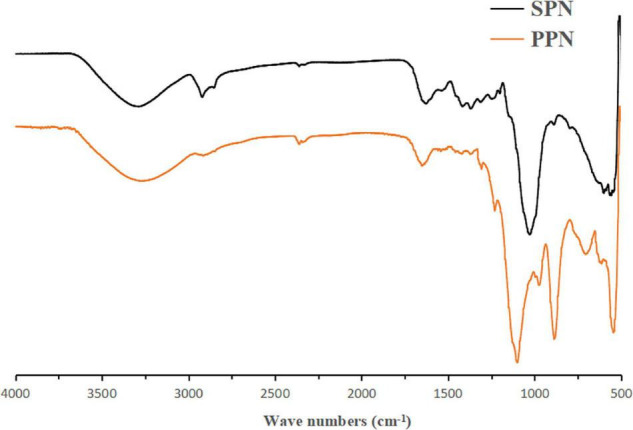
FT-IR spectra of SPN and PPN.

In the FI-IR spectrum (Figure 2), the wide and big absorption peak around 3,400 cm^–1^ was the characteristic absorption peak of O-H which determined SPN and PPN were polysaccharides (44). Compared to SPN, after substitution of phosphate group, O-H absorption peak of PPN was weaker which indicated that intermolecular hydrogen bonding action of PPN was weaker than that of SPN. The absorption peak between 1,400 and 1,200 cm^–1^ was variable angle vibration of C-H. However, in PPN spectrum, this absorption peak shifted from 1,361 to 1,305 cm^–1^ because of phosphorylation. The absorption peaks of SNP at 895, 1,025 cm^–1^, and PPN at 895 cm, 1,023 cm^–1^ indicate that they are pyranose containing β configuration. The absorption peaks of SPN at 1,639, 1,423 cm^–1^ and PPN at 1,647, 1,417 cm^–1^ indicated that they were acidic polysaccharides, which was consistent with the determination of uronic acid (45). Also, the new absorption peak of 1,241 cm^–1^ was the asymmetrically stretching vibration of P = O (46) and the new peaks around 727 cm^–1^ attributed to C-O-P symmetrically stretching vibration compared to spectrum of SPN (47), but all peaks were not obvious because the lower degree substitution of phosphate group. FI-IR spectra showed phosphorylation of SPN changed its structure and probably would have effect on its biological function.

According to the standard dextran of different molecular weight (logMw = -0.3991RT + 17.78, *R*^2^ = 0.9968), the average molecular weight of SPN was 15.8 kDa with the retention time of 34.03 min and the average molecular weight of PPN was 27.7 kDa with the retention time of 33.42 min. In addition, both elution peaks were single and symmetric in the GPC spectra, indicating they were both homogeneous fractions (48).

HPLC analysis shown in Table 2 determines the monosaccharide composition of SPN and PPN. They both mainly contained rhamnose, glucose, arabinose, galacose, and mannose. However, the contents of each monosaccharide in the two polysaccharides were different as previous research reported (49). SPN contained 7.4% rhamnose, 6.4% mannose, 38.6% glucose, 20.5% arabinose, and 27.1% galacose, while PPN contained 8.5% rhamnose, 7.3% mannose, 44.9% glucose, 15.7% arabinose, and 23.6% galacose by area ratio. The results showed that phosphorylation increased the ratio of glucose and arabinose, while decreased the ratio of galactose.

**TABLE 2:** Monosaccharide analysis data.

Compositions	SPN content (%, area)	PPN Content (%, area)
Mannose	6.4	7.3
Glucose	38.6	44.9
Galacose	27.1	23.6
Arabinose	20.5	15.7
Rhamnose	7.4	8.5

### Methylation analysis

In order to analyze the glycosyl linkages of polysaccharides, methylation method with GC-MS was used. After two times of methylation, the characteristic absorption peak of O-H around 3,400 cm^–1^ was almost disappeared in the FI-IR spectra of methylated samples, indicating the methylation of samples were completed.

Form Table 3, the mainly methylated sugars of SPN contained 2,3,4,6-Me_4_-Glc*p*(8.4%), 2,3,6-Me_3_-Glc*p*(34.5%), 2,3,4-Me_3_-Gal*p*(16.7%), 2,3-Me_2_-Gal*p*(7.9%), 2,3,5-Me_3_-Ara*f*(6.6%), 3-Me-Ara*f*(5.5%), 2,3-Me_2_-Ara*f*(10.9%), 3,5-Me_2_-Rha*p*(5.0%) and 2,3,4-Me_3_-Man*p*(4.5%), PPN contained 2,3,4,6-Me_4_-Glc*p*(12.7%), 2,3,6-Me_3_-Glc*p*(21.2%), 2,3,4-Me_3_-Gal*p*(13.5%), 2,3-Me_2_-Gal*p*(9.5%), 3,6-Me_2_-Glc*p*(13.5%), 2,4,6-Me_3_-Gal*p*(4.1%), 2,3,5-Me_3_-Ara*f*(11.4%), 3,5-Me_2_-Rha*p*(6.2%), and 2,3,4-Me_3_-Man*p*(7.9%). The relative proportions of terminal residues and branching residues were almost equal, indicating that the polysaccharides were completely methylated (50). In addition, the proportion of glycosyls in methylation analysis was consistent with that in monosaccharide composition. However, the disappeare of 1,2,5-linked Ara*f*, 1,5-linked Ara*f* and the generation of 1,2,4-linked Glc*p*, 1,3-linked Gal*p* in PPN indicated that they were located in the branched chain. The man chains of the SPN and PPN were composed of 1,4-linked Glc*p*, 1,6-linked Gal*p*, 1,2-linked Rha*p*, 1.6-linked Man*p* and the terminals of t-linked Glc*p*, t-linked Ara*f*. This result suggested that phosphorylation did not change the main chain structure of the polysaccharide. The side chain of SPN is 1,4,6-linked Gal*p*, 1,2,5-linked Ara*f*, while PPN is 1,4,6-linked Gal*p*, 1,2,4-linked Glc*p*. It is shown that the phosphorylation changes the branching structure of the polysaccharide. This might be due to the different effects of phosphorylation on the hydrolysis of different glycosidic bonds. For example, the branched chains are easier hydrolyzed than the backbone, and furanose is more hydrolyzed than pyranose (51).

**TABLE 3:** Methylation analysis data.

Methylated sugars	Linkage patterns	SPN relative amount (%, area)	PPN relative amount (%, area)
2,3,4,6-Me_4_-Glc*p*	1-linked Glc*p*	8.4	12.7
2,3,6-Me_3_-Glc*p*	1,4-linked Glc*p*	34.5	21.2
3,6-Me_2_-Glc*p*	1,2,4-linked Glc*p*	–	13.5
2,3,4-Me_3_-Gal*p*	1,6-linked Gal*p*	16.7	13.5
2,3-Me_2_-Gal*p*	1,4,6-linked Gal*p*	7.9	9.5
2,4,6-Me_3_-Gal*p*	1,3-linked Gal*p*	–	4.1
2,3,5-Me_3_-Ara*f*	1-linked Ara*f*	6.6	11.4
3-Me-Ara*f*	1,2,5-linked Ara*f*	5.5	–
2,3-Me_2_-Ara*f*	1,5-linked Ara*f*	10.9	–
3,5-Me_2_-Rha*p*	1,2-linked Rha*p*	5.0	6.2
2,3,4-Me_3_-Man*p*	1.6-linked Man*p*	4.5	7.9

### NMR analysis

**FIGURE 3:**
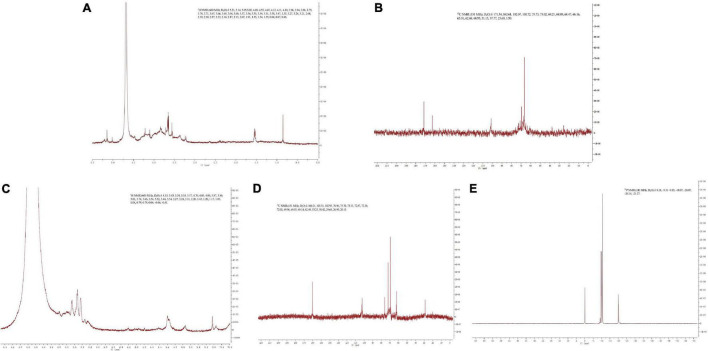
^1^H NMR spectra of SPN **(A)** and PPN **(C)**, ^13^C NMR spectra of SPN **(B)** and PPN **(D)**, and ^31^P NMR spectra of PPN **(E)**.

The NMR spectrum of SNP and PPN are shown in Figure 3. In ^1^H NMR, the chemical shift range of heterocephalic hydrogen signal from SNP and PPN was less than 5 ppm, which indicated that they contained β configurations (52). At the same time, this conclusion was proved by 102–112 ppm chemical shift peaks of the polysaccharides in ^13^C NMR (53). The absence of signal peak at 5.40 ppm indicated that PPN and SPN were pyranose, which was consistent with the results of the FI-IR. ^13^C NMR signals of PPN and SPN were mainly at 170–20 ppm. The signals of PPN at 103.51, 75.70, 73.33, 72.02, 69.55, and 62.49 ppm and SPN at 102.07, 75.73, 73.02, 69.21, 68.47, and 66.16 ppm were attributed to C1–C6 carbon atoms. The shift of chemical from 62.49 to 67 ppm indicated that substitution might take place at C-6 (54). In addition, the signal peak at 167 ppm was a chemical shift peak caused by phosphate substitution. Due to the insufficient solubility of PPN in D_2_O, the carbon spectrum needs to be further studied. The significant absorption peaks at 0.31, –9.83, –10.07, and –20.07 ppm of PPN were shown in the ^31^P NMR, which indicated phosphorylation substitution reactions occurred at multiple positions from the sugar ring (55). This is consistent with previously published results by Liu and Huang (56).

### SEM analysis

**FIGURE 4:**
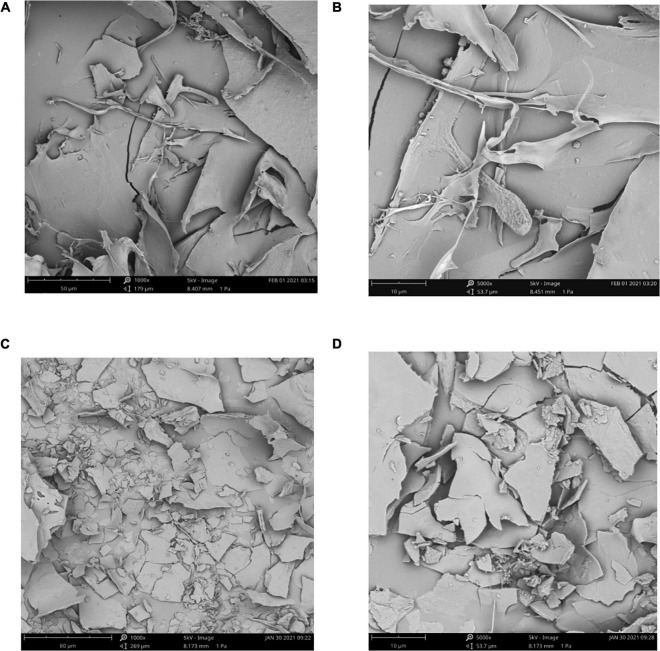
SEM spectra of SPN (1000×) **(A)**, SPN (5000×) **(B)**, PPN (1000×) **(C)**, and PPN (5000×) **(D)**.

In SEM spectra (Figures 4A,B), it showed smoothly large chunks of debris with a network on surface of SPN, which indicated that the structure of SPN is tight. However, in SEM spectra of PPN (Figures 4C,D), there were many cracks and small debris on the surface of PPN which meant the structure of PPN is loosen and multi-level, probably due to the phosphorylation increased the distance between molecules of PPN. The structure of PPN is more conducive to the entry of water, which increases the solubility and affects the biological activity (57).

### Antioxidant activity analysis

#### DPPH radical scavenging activity

**FIGURE 5:**
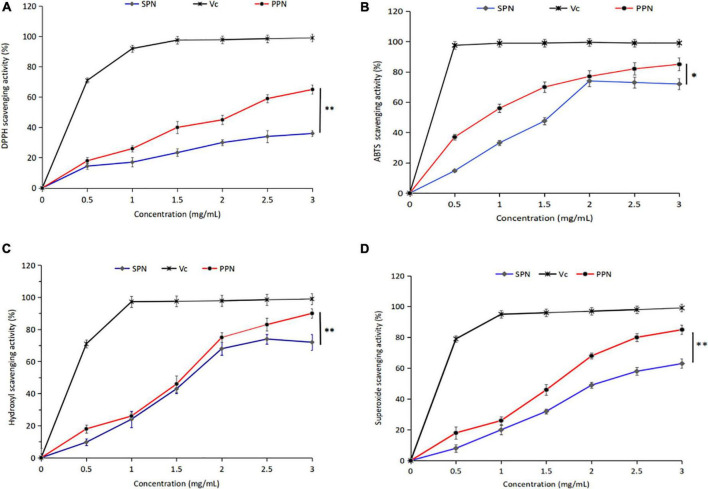
DPPH radical scavenging activity **(A)**, ABTS radical scavenging activity **(B)**, Hydroxyl scavenging activity **(C)**, and Superoxide scavenging activity **(D)** of PPN and SPN. *Indicates *P* < 0.05, **indicates *P* < 0.01.

DPPH is a stable-free radical with three benzene rings, DPPH radical scavenging method has the characteristics of easy operation and fast reaction. Figure 5A shows the effect of SPN and PPN on DPPH radical scavenging activity at 0–3 mg/mL. With the increase of concentration, the DPPH radical scavenging rate of SPN and PPN increased, presenting a certain dose-dependence. At the concentration of 3 mg/mL, the radical scavenging rates of SPN and PPN were 37.8 and 65.9%, respectively, indicating that phosphorylated polysaccharides had significantly better DPPH antioxidant activity than natural polysaccharides (*p* < 0.01), but weaker than Vc.

#### ABTS radical scavenging activity

ABTS radical scavenging method is a common method for the determination of antioxidant activity, which has the advantage of substrate color without interference. From Figure 5B, the ABTS radical scavenging rate escalated with the increase of PPN concentration at 0–3 mg/mL. However the scavenging activity decreased, when the concentration of SPN exceeded 2 mg/mL. The ABTS radical scavenging rate of SPN and PPN were 67.1 and 86.4% at polysaccharide concentration of 3 mg/mL, the difference was significant (p < 0.05). The ABTS radical scavenging activity of PPN was higher than SPN and close to Vc.

#### Hydroxyl radical scavenging activity

Hydroxyl-free radical is a kind of reactive oxygen species, which can react to cellular components, resulting in harm to the body. From Figure 5C, the hydroxyl radical scavenging rate of SPN and PPN ascended with the increase of PPN concentration at 0–3 mg/mL. At polysaccharide concentration of 3 mg/mL, the ABTS radical scavenging rate of SPN and PPN were 78.3 and 93.6%, respectively. PPN has significantly stronger scavenging activity than SPN (*p* < 0.01), and slightly weaker than Vc. Chen and Huang (43) reported that the phosphorylated pumpkin polysaccharide had better free radical scavenging activity than natural polysaccharide.

#### Superoxide radical scavenging activity

Superoxide-free radical is a kind of reactive oxygen species produced in human body, which can cause lipid peroxidation and accelerate the aging process of the body. From Figure 5D, SPN and PPN had superoxide radical scavenging activity, and their radical scavenging rate were 63.5 and 86.7%, respectively, at concentration of 3 mg/mL, indicating phosphorylation significantly enhanced antioxidant activity (*p* < 0.01). This conclusion is recognized by Chen and Huang (46).

### Inflammatory effect on RAW 264.7 cells

#### Cell viability of RAW 264.7 cells

**FIGURE 6:**
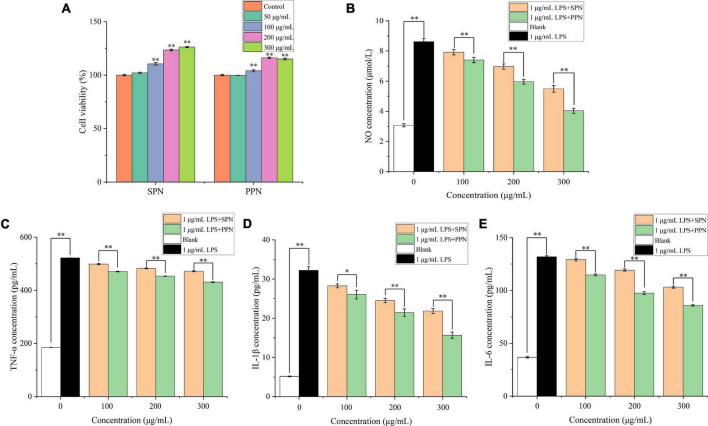
Cell viability **(A)**, NO concentration **(B)**, TNF-α concentration **(C)**, IL-1β concentration **(D)** and IL-6 concentration **(E)** of RAW 264.7 cells. *Indicates *P* < 0.05, **indicates *P* < 0.01.

The cell viability of RAW 264.7 cells treated by different concentrations of SPN and PPN was evaluated by CCK-8 commercial kit. In Figure 6A, SPN and PPN of 50 μg/mL had no significant influence on viability of RAW 264.7 cells compared to control group, while SPN and PPN above 50 μg/mL can significantly increase viability of RAW 264.7 cells which indicated both polysaccharides above 50 μg/mL can promote RAW 264.7 cells proliferation. SPN and PPN are non-toxic to macrophages. Therefore, the other three concentrations (100, 200, and 300 μg/mL) were selected for following experiments.

#### NO concentration analysis

NO is a key cytokine that mediates inflammatory responses and is widely involved in a variety of physiological processes *in vivo*. Previous research proved that NO concentration is an important index to evaluate macrophage activation (58). In Figure 6B, the NO concentration in blank control group was 3.1 μmol/L, while RAW 264.7 cells released great amount of NO after 1 μg/mL LPS treatment for 24 h (8.6 μmol/L). Compared with blank control group, the NO concentration of LPS treatment group extremely increased (*p* < 0.01). However, with different concentrations of polysaccharides and LPS treatments for 24 h, the NO levels significantly reduced (*p* < 0.01), and the reducing effects were more significant in higher concentration polysaccharides treatments, compared with LPS treatment group. Besides, the reducing effect of NO concentration by PPN was better than that by SPN in same concentration. This is consistent with previously published results by Yang et al. (59).

#### TNF-α, IL-1β, and IL-6 levels analysis

TNF-α, mainly produced by monocytes, macrophages, has functions of inflammatory regulation, participation in fever and inflammation occurrence (60). In Figure 6C, after 24 h treatment with 1 μg/mL LPS, TNF-α level significantly raised from 184.9 to 521.8 pg/mL (*p* < 0.01), compared with blank control group. Similar with NO concentration results, TNF-α level significantly decreased with polysaccharides concentration increasing (*p* < 0.01), and the effect of SPN was always weaker than that of PPN in same concentration. This result was also identified by Tian et al. (61).

IL-1β, mainly released by activated macrophages, can play a role in inflammatory regulation (62). In Figure 6D, the IL-1β level of LPS model group extremely increased from 5.2 to 32.2 pg/mL compared to blank control group (*p* < 0.01). Also, the IL-1β level of polysaccharides treatment groups gradually decreased as concentration of polysaccharides increased. Moreover, the decreasing effect on IL-1β concentration of PPN was always better than that of SPN.

IL-6 is one of the most common pro-inflammatory cytokines secreted mainly by activated macrophages, lymphocytes, and epithelial cells, and has multiple inflammatory functions, which can participate in vivo inflammatory response as an important inflammatory medium (63). From Figure 6E, 1 μg/mL LPS treatment for 24 h can significantly enhance the IL-6 level from 36.7 to 131.8 pg/mL compared with blank control group (*p* < 0.01). With the increase of polysaccharides concentration, IL-6 level decreased gradually. In addition, the ability of inhabiting IL-6 release of PPN was always better than that of SPN in every concentration.

#### mRNA expression levels analysis

**FIGURE 7:**
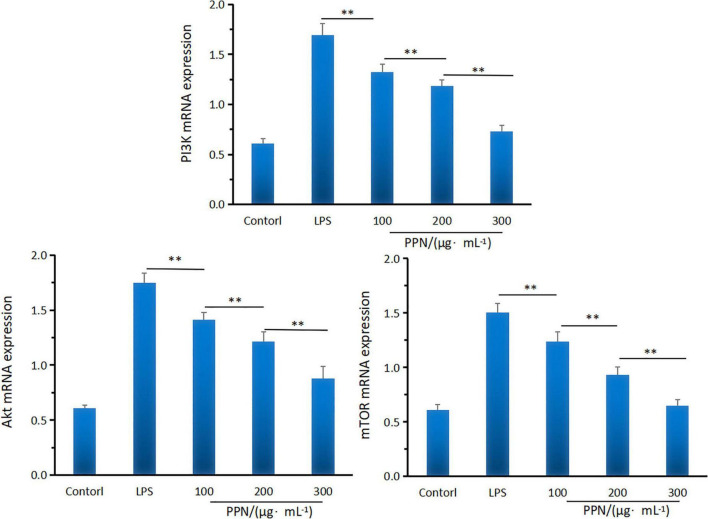
The PI3KAktmTOR mRNA expression of SPN and PPN. **Indicates *p* < 0.01.

Quantitative RT-PCR was used to investigate the effect of PPN on the mRNA expression of PI3K, Akt, and mTOR. From Figure 7, 1 μg/mL LPS treatment for 24 h can significantly promote the mRNA expression of PI3K, Akt, and mTOR. Compared with the LPS model group, PPN significantly inhibited the mRNA expression of PI3K, Akt, and mTOR (*p* < 0.01). The mRNA expression level of higher concentration polysaccharide group was significantly higher than that of other concentration group (*p* < 0.01). The level of mRNA expression in cells was dose-dependent with the PPN concentration. The results showed that PPN played an anti-inflammatory role by down regulating the mRNA expression of PI3K, Akt, and mTOR. More data could see the Supplementary material.

#### Protein expression levels analysis

**FIGURE 8:**
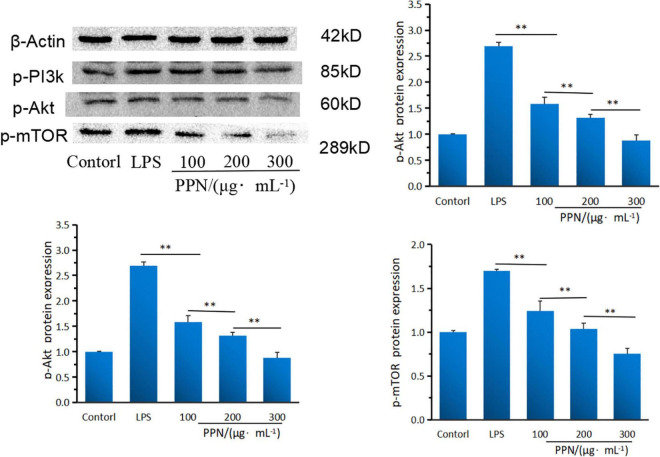
The PI3KAktmTOR protein expression of SPN and PPN. **Indicates *p* < 0.01.

Western blot was used to analyze the effect of PPN on key protein expression of PI3K/Akt/mTOR pathway. From Figure 8, compared with LPS model group, PPN groups significantly inhibited the protein expression of p-pI3k, p-Akt and p-mTOR (*p* < 0.01). The protein expression level of high-dose polysaccharide group was significantly lower than that in low-dose group (*p* < 0.01). And the protein expression level was dose-dependent with PPN concentration. PPN could down-regulate p-PI3K expression level, blocking PI3K/AKT signal, decreasing phosphorylation AKT protein level, leading to the decrease expression level of p-mTOR. Thereby the secretion of TNF-α, IL-1β, IL-6 were down regulated which reduced the inflammatory reaction. In conclusion, PPN can regulate inflammatory cytokines through PI3K/Akt/mTOR pathway. From structural analysis of SPN and PPN, it is obvious that phosphorylation has changed the structure of SPN. Apparently, due to the structure changed, the biological function of SPN and PPN are different. In antioxidant activity experiments, the free radical scavenging rate of PPN was significantly stronger than that of SPN (*p* < 0.05). In cell model experiments, PPN always has significantly better anti-inflammatory effect on RAW 264.7 cells induced by LPS than SPN (*p* < 0.01), especially at same concentration. After substitution of phosphate group, phosphate group replaced some hydroxy groups on original chain of SPN, resulting in changes on its structure which made more hydroxy groups expose (64, 65). In addition, the changes of molecular weight, monosaccharide ratio, and molecular configuration of PPN might affect its biological activity (66). Thus, the biological function of phosphorylated polysaccharide improved.

PI3K/Akt/mTOR pathway is one of the important signaling pathways of the inflammatory response, which can regulate the activation of signal kinase and the expression of downstream gene and proteins to inhibit the inflammatory response of cells (67, 68). In this study, the potential anti-inflammatory mechanism of PPN was analyzed by the gene and protein expression levels of PI3K, Akt and mTOR. PI3K is an inositol kinase and act as a Biomarker to regulate cell differentiation, proliferation and apoptosis. PPN significantly down-regulated the PI3K gene and protein expression levels (*p* < 0.01). Akt is an important downstream factor of PI3K, and lower p-PI3K protein level could lead to Akt phosphorylation level drop. This indicated that PPN blocked PI3K/Akt pathway signals and reduced the protein expression level of p-Akt at threonine 308 site of Akt, which lead to the blocking of p-Akt downstream signal. mTOR (mammalian target of rapamycin) is a downstream target of Akt. The protein expression of p-Akt was blocked, resulting in the inhibition of mTOR activity. Collectively, PPN can down-regulate the secretion level of TNF-α, IL-1β and IL-6 *via* PI3K/Akt/mTOR pathway. However, the structure-function relationship of phosphorylated polysaccharide and the anti-inflammatory interaction of other pathways are still unclear, which need further research.

## Conclusion

In this study, we analyzed the structural, antioxidant activity, and anti-inflammatory effect differences of SPN and PPN. SPN and PPN were β-pyranose configuration and mainly contained glucose, galacose, and arabinose. Phosphorylation increased the molecular weight of polysaccharide from 15.8 to 27.7 kDa. FI-IR, NMR, and SEM spectra showed phosphorylation of SPN changed its structure and confirmed that the chemical modification was successful. Besides the phosphorylation did not change the main chain structure but changed the branched chain structure by methylation. Moreover, PPN always has better antioxidant activity and anti-inflammatory than SPN. PPN can inhibit inflammatory by PI3K/AKT/mTOR signal pathway. These findings are helpful to study the structural characterization, antioxidant activity and anti-inflammatory effect of phosphorylated *pholiota nameko* polysaccharide by high-temperature pressurized extraction.

## Data availability statement

The original contributions presented in this study are included in the article/Supplementary material, further inquiries can be directed to the corresponding author.

## Author contributions

XZ: conceptualization, methodology, and writing—original draft. LZ: investigation and resources. JQ: formal analysis and visualization. XW: resources and formal analysis. SA: conceptualization and methodology. TL: resources, data curation, and supervision. All authors contributed to the article and approved the submitted version.
