# Supplementary figures and images for: Structural characterization, antioxidant activity and anti-inflammatory of the phosphorylated polysaccharide from Pholiota nameko
Source: Front Nutr. 2022 Aug 31;9:976552. doi: 10.3389/fnut.2022.976552 (PMC9471013; doi:10.3389/fnut.2022.976552)

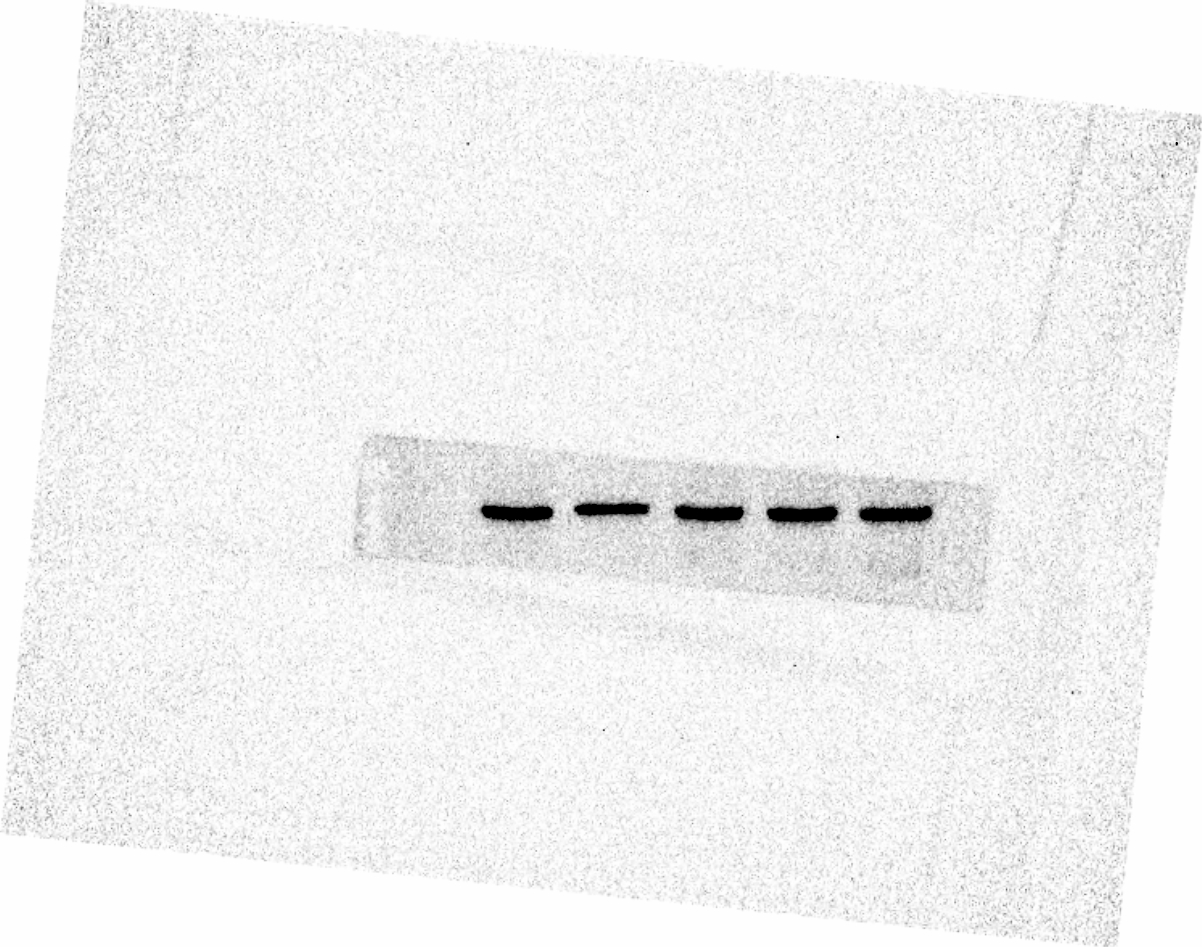

Supplement: Supplementary file 1 [file Data_Sheet_1.ZIP › Additional files/WB/actin.jpg]

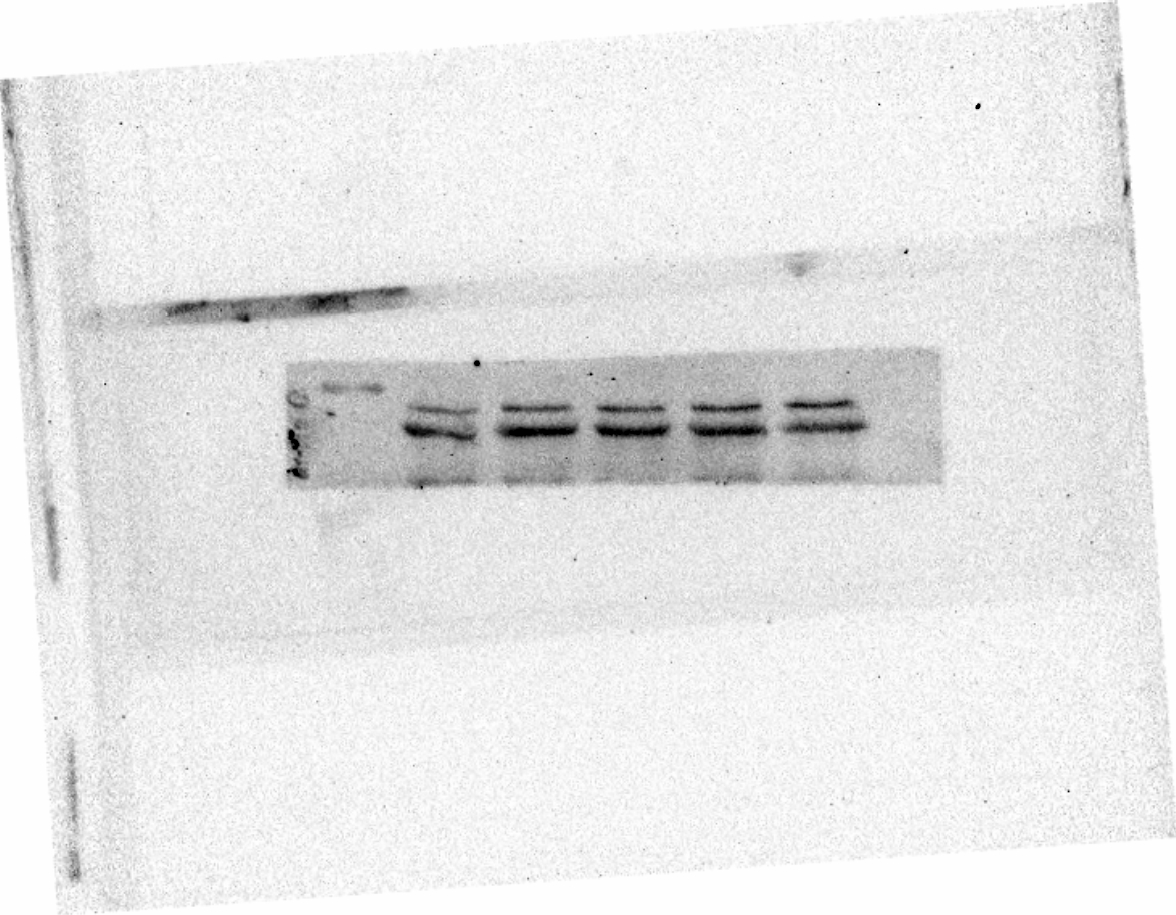

Supplement: Supplementary file 1 [file Data_Sheet_1.ZIP › Additional files/WB/p-Akt .jpg]

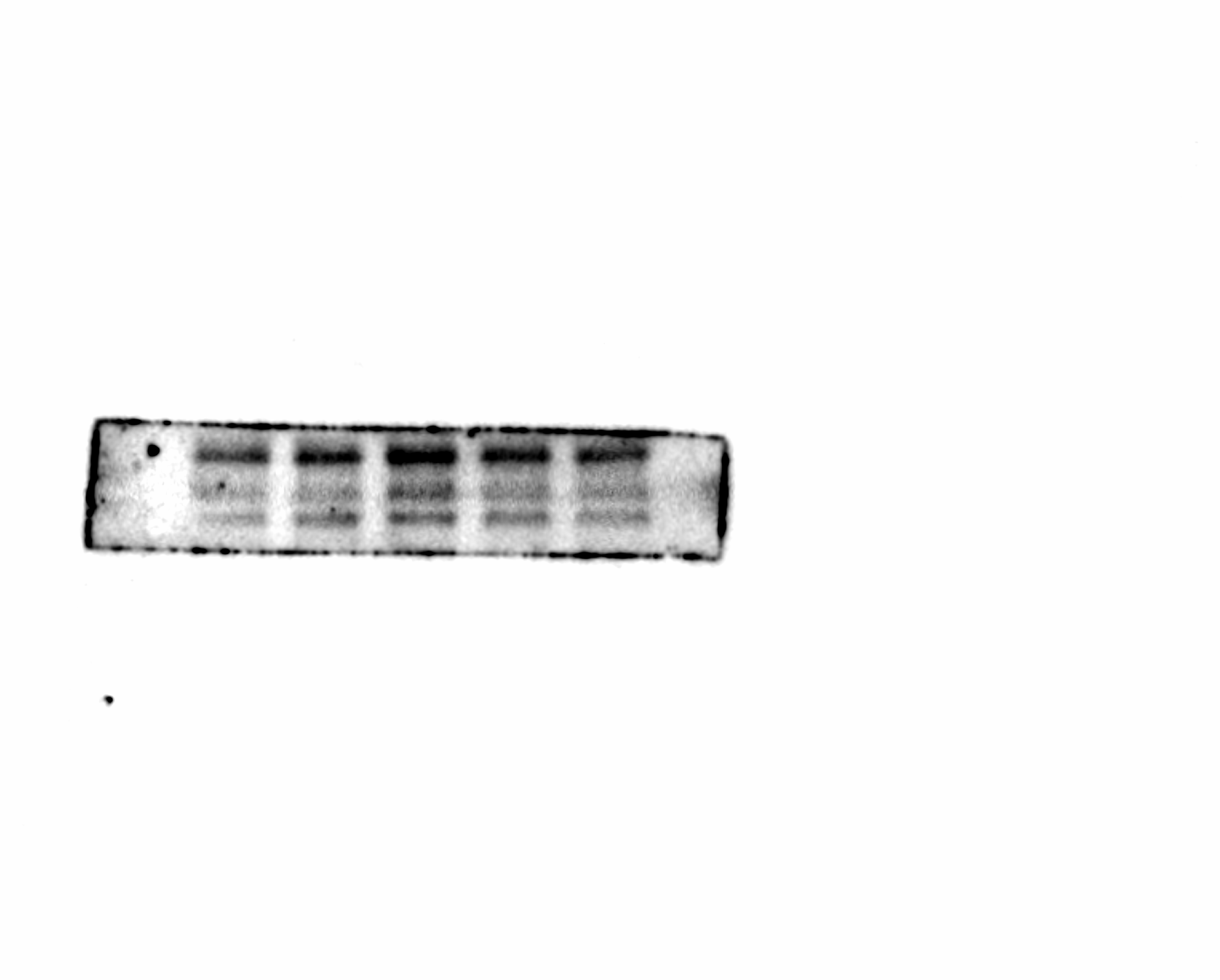

Supplement: Supplementary file 1 [file Data_Sheet_1.ZIP › Additional files/WB/p-PI3k.jpg]

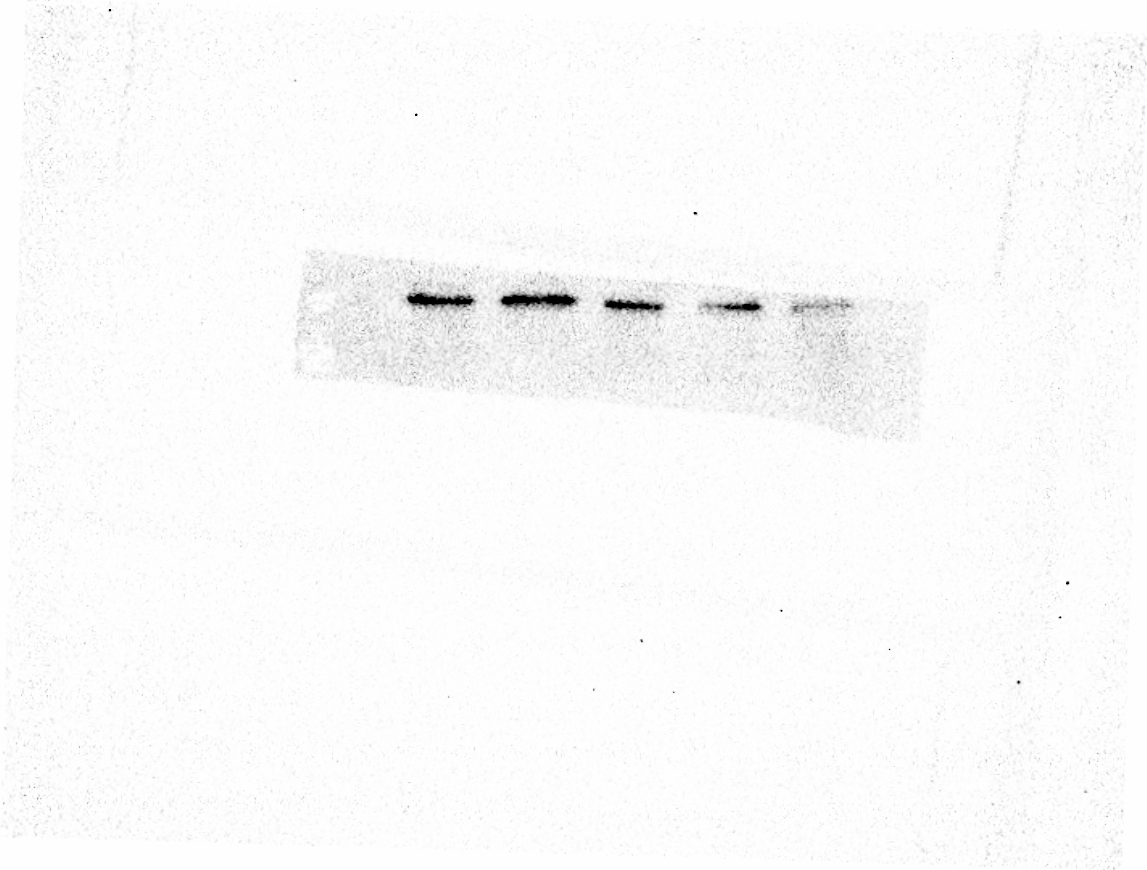

Supplement: Supplementary file 1 [file Data_Sheet_1.ZIP › Additional files/WB/p-mTOR .jpg]
